# Antiviral Potential of *Momordica charantia*: From Traditional Use to Modern Implications

**DOI:** 10.3390/biomedicines14020412

**Published:** 2026-02-11

**Authors:** Massimo Bortolotti, Francesco Biscotti, Andrea Bolognesi, Letizia Polito

**Affiliations:** Department of Medical and Surgical Sciences—DIMEC, Alma Mater Studiorum, University of Bologna, Via San Giacomo 14, 40126 Bologna, Italy; massimo.bortolotti2@unibo.it (M.B.); francesco.biscotti2@unibo.it (F.B.)

**Keywords:** antiviral activity, bitter melon, folk medicine, MAP30, *Momordica charantia*, momordins, ribosome-inactivating proteins, triterpenoids

## Abstract

**Background/Objectives**: *Momordica charantia* L. (Cucurbitaceae) (MC), commonly known as bitter melon, is a prominent therapeutic and edible species deeply rooted in traditional medicine for the management of diverse metabolic and infectious pathologies. Increasing evidence suggests that MC is a significant source of antiviral compounds that could act against many different types of viruses in humans. This narrative review summarizes the current knowledge regarding the antiviral properties of MC, with a focus on molecular mechanisms and therapeutic perspectives. **Methods**: A comprehensive literature search was conducted across the PubMed, Scopus and Web of Science databases, using the keywords “*Momordica charantia*”, “bitter melon”, “antiviral” and “MAP30”. Original in vitro, in vivo, in silico and mechanistic studies were included. **Results**: MC harbors diverse antiviral molecules acting through conserved and virus-specific mechanisms. Ribosome-inactivating proteins (RIPs) purified from MC display potent antiviral activity by catalyzing the depurination of viral RNA and DNA, inactivating host ribosomes and blocking protein synthesis. RIPs, especially MAP30, are among the most potent natural antiviral proteins described to date. Cucurbitane-type triterpenoids and other phytochemicals from MC also show antiviral activity. **Conclusions**: MC emerges as a promising candidate for the prevention/treatment of viral diseases through nutraceutical, topical and pharmaceutical applications. MC extracts could represent a resource to support the immune system and provide broad-spectrum benefits against viral infections or a tool for local treatments. Moreover, MC is a valuable source of various bioactive compounds that, after thorough pharmacological characterization, could be further developed into specific antiviral agents.

## 1. Introduction

Viral infections pose a serious threat to global health, despite the availability of vaccines and several classes of antiviral drugs [1]. Historically, medicinal plants have been an important source of antiviral molecules, providing structurally complex compounds that can affect multiple stages of the viral life cycle [2].

**Figure 1:**
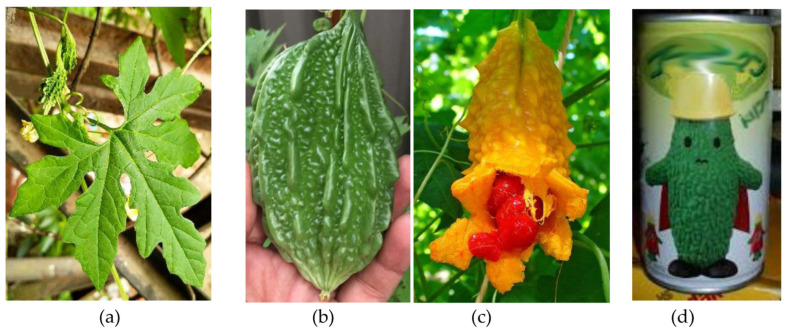
Different parts and a cultural representation of *Momordica charantia* (bitter melon or “goya”). (**a**) The early growth stage of the plant, showing deeply lobed leaves; (**b**) an unripe green fruit with its characteristic warty surface; (**c**) a fully ripened fruit, yellow-orange in color, naturally dehiscent to expose bright red arils surrounding the seeds; and (**d**) a commercial beverage product from Japan featuring a cartoon character, “Goya-man”, illustrating the integration of bitter melon into popular culture and consumer goods.

Amongst these medicinal plants, a significant role is represented by plants from the Cucurbitaceae family and, in particular, by *Momordica charantia* L. (MC), commonly known as bitter gourd, balsam pear, bitter melon, kugua or karela [3,4]. Its name derives from the Latin verb *momordi* (to bite), referring to the leaves, which look to have been bitten (Figure 1a), and from the Greek noun *chárax*, meaning “support cane”, for its use as a climber in pergolas. MC is widely cultivated in tropical and subtropical regions, mainly for its wide uses in folk medicine and for culinary purposes [5,6]. The most commonly used parts of the plant are the seeds and fruits, which appear spindle-shaped with pimples on the surface, looking like a small cucumber; unripe fruits are emerald green in color, which turns to orange during ripening (Figure 1b,c). In many parts of the world, MC fruits are eaten as vegetables, despite their bitter taste. This widespread consumption is mainly due to bitter melon’s high nutritional value and numerous bioactive effects [7]. Nowadays, in different Asian regions, mainly China, India and Thailand, several bitter gourd products are quite popular; for example, bitter gourd tea, known as gohyah, or soft drinks are commonly consumed (Figure 1d).

Numerous medicinal properties of MC have been investigated, particularly its hypoglycemic, antibacterial, anti-inflammatory, antioxidant and anti-tumor activities [8,9,10,11,12,13,14]. In addition, several in vitro studies have confirmed that bitter melon possesses inhibitory effects against different human viruses. These beneficial effects are attributed to the various bioactive components of MC, which are important sources of phytoconstituents used to treat various diseases since ancient times [15,16,17]. While the aforementioned therapeutic activities have been well-documented and experimentally supported [6], antiviral research on MC is still fragmented. Most studies have focused on individual compounds or crude extracts, often using different experimental conditions, viral strains and outcome measures. Standardized preparations and comparative studies are quite scarce and clinical data are lacking.

This narrative review aims to (i) describe the role of *Momordica charantia* L. (Cucurbitaceae) in folk medicine as an antiviral remedy; (ii) summarize the phytochemical constituents of MC relevant to antiviral activity and describe the spectrum of antiviral effects reported against distinct viruses; and, finally, (iii) detail the antiviral activity of ribosome-inactivating proteins (RIPs) in MC. A comprehensive literature search was conducted across the PubMed, Scopus and Web of Science databases. The search strategy employed combinations of the keywords “*Momordica charantia*”, “bitter melon”, “antiviral” and “MAP30”. The search included articles published from 1986 to 2025. Studies were selected based on the following inclusion criteria: (1) original research articles including in vitro, in vivo and in silico models and (2) studies specifically investigating molecular mechanisms of *Momordica charantia* derivatives against viruses. Studies focusing solely on the plant’s nutritional or agricultural aspects, without considering antiviral properties, were excluded from this review.

## 2. *Momordica charantia* in Folk Medicine as Antiviral Remedy

MC is widely used as a medicinal and food plant across Asia, Africa, the Caribbean and Latin America. Although pre-modern medical systems did not use the term “virus/viral”, ethnomedical uses of MC commonly target syndromes, such as fever, respiratory complaints and eruptive skin conditions, that are today often attributable to viral infections. Ethnobotanical records document local uses of MC as a household remedy for such conditions, providing consistent cultural evidence of its perceived antiviral role [18].

In India and South Asian neighboring regions, folk and regional plant compendia list MC leaf decoctions, fruit juices and topical pastes as remedies for febrile illnesses, sore throat, cough and measles-like eruptions. These preparations are used both curatively and prophylactically during seasons of increased illness [19]. Leaf infusions and fruit-based teas are taken for “fever”, flu-like syndromes and childhood eruptive diseases. Moreover, crushed leaves or fruit pulp are topically applied to skin lesions [20]. In Chinese folk practice, popular uses of bitter melon include intake for “clearing heat” and topical use for ulcerative or vesicular skin conditions—uses aligned with the treatment of febrile and eruptive illnesses of presumed infectious origin [19].

Ethnopharmacological studies in West Africa explicitly document MC as a traditional remedy for febrile diseases, respiratory complaints and skin conditions resembling measles or other eruptive illnesses. Data from Togo (village interviews and surveys) report oral use (leaf decoctions) for fevers and topical use for eruptive lesions. These uses are explicitly connected by informants to contagious febrile conditions [21].

In Caribbean folk medicine, bitter melon is widely used as a household remedy (teas, decoctions, and poultices) for fever, influenza-like illness, sore throat and eruptive rashes [3]. In Trinidad and Tobago, household uses of bitter herbs (including bitter melon) have been reported for the symptomatic relief of febrile and “virus-like” conditions [22].

Across regions, the antiviral use of MC in folk medicine shows recurring patterns, such as (i) oral febrifuge/respiratory remedies—leaf or fruit decoctions/infusions used at the onset of fever or cough; (ii) topical treatment of eruptive lesions—crushed leaves/fruit pulp applied to vesicular or ulcerative rashes; and (iii) prophylactic household use—teas consumed routinely during outbreak seasons. These consistent ethnomedical motifs across independent cultural contexts form the basis for describing MC as a widely used folk antiviral remedy, with the understanding that ethnobotanical use does not mean proven clinical efficacy.

## 3. Phytochemistry of *Momordica charantia*

MC has a rich and heterogeneous phytochemical profile, including numerous substances distributed throughout almost all plant organs and tissues (fruit, seeds, leaves, pericarp and roots). The principal classes of phytochemicals include (i) cucurbitane-type triterpenoid glycosides (e.g., momordicosides and karavilosides); (ii) RIPs such as momordica anti-HIV protein 30 kD (MAP30) and momorcharins; (iii) polysaccharides; (iv) flavonoids, phenolic acids and saponins (quercetin/kaempferol glycosides, catechins, gallic acid, and charantin); and (v) sterols (β-sitosterol, stigmasterol, and linoleic/oleic acids) [9,23]. Among these, RIPs are the most strongly associated with antiviral effects, while the other constituents appear to have mainly immunomodulatory effects supporting antiviral activity (Table 1).

### 3.1. Cucurbitane-Type Triterpenoid Glycosides

**Figure 2:**
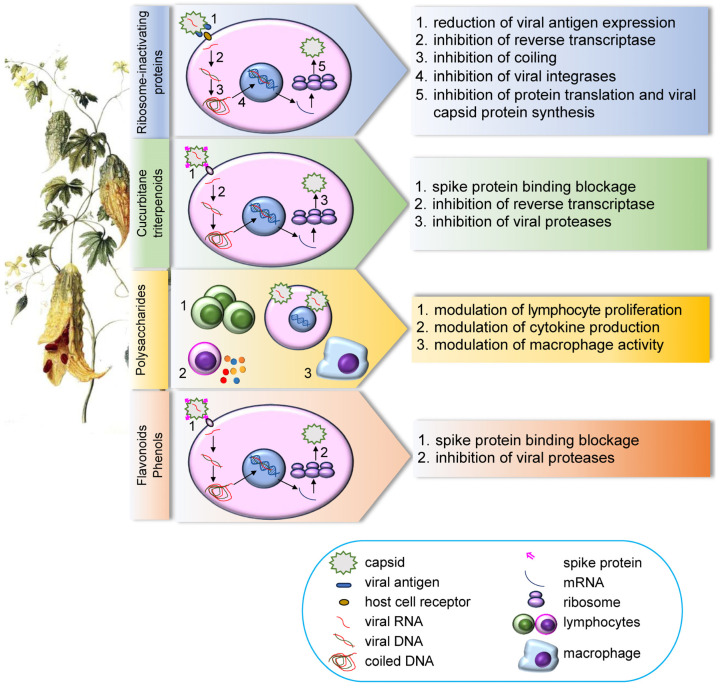
The various classes of *Momordica charantia* antiviral compounds exert their activity through several mechanisms involving different molecular and cellular targets.

Cucurbitane-type triterpenoid glycosides are among the most distinctive metabolites of MC and have been investigated for their antiviral potential. Their structural diversity, including that of momordicosides and karavilosides, has been linked to interference with viral enzymes and replication pathways (Figure 2).

Extracts obtained from the vines and leaves of bitter melon led to the isolation of fourteen cucurbitane triterpenoids, designated kuguacins F–S. These compounds were tested in vitro against human immunodeficiency virus-1 (HIV-1)-infected cells; several of them showed weak antiviral activity, with EC_50_ values ranging from 3.7 to 61 µg/mL and selectivity indices (i.e., the ratios between the concentration killing 50% of host cells and the concentration inhibiting viral replication by 50%) of up to 13.3. Although they were less potent than the reference drug azidothymidine, these cucurbitane derivatives were shown to directly inhibit HIV-1 replication [25].

Other cucurbitane-type triterpenoids extracted from the roots of MC were tested for in vitro inhibitory effects against HIV replication in C8166 cells. Kuguacin C and E displayed moderate anti-HIV-1 activity, with EC_50_ values of 8.45 and 25.62 µg/mL, and exerted minimal cytotoxicity against C8166 cells (IC_50_ > 200 µg/mL), with selectivity index values greater than 23.68 and 7.81, respectively [28].

Further investigations confirmed inhibition of HIV-1 reverse transcriptase and protease using ELISA-based assays. Complementary molecular docking analyses revealed strong binding affinities of momordicosides to the active sites of these enzymes, providing both biochemical and mechanistic support for the antiviral potential of cucurbitane glycosides [24].

Multiple docking and molecular modeling studies have identified MC triterpenoids (e.g., momordicine I and related cucurbitanes) as candidate binders of severe acute respiratory syndrome coronavirus 2 (SARS-CoV-2) targets (spike protein or main protease) and other viral enzymes. These in silico results generate testable hypotheses but require biochemical and cell-based validation [36].

Recently, computational studies and docking simulations have suggested possible effects of cucurbitane glycosides from several plants but not MC on the influenza virus, indicating stable theoretical interactions with neuraminidase and leading to hypotheses of potential inhibitory effects against viral replication, which await confirmation through in vitro assays [37]. These results suggest a wider range of antiviral activity, encouraging further research on MC compounds supported by experimental validation.

In silico analysis showed that karaviloside III has strong inhibitory activity and that momordicoside B, kuguaglycoside A and cucurbitadienol also show inhibitory actions against the spike glycoprotein, main protease and RNA-dependent RNA polymerase of SARS-CoV-2, suggesting that these compounds represent candidates for further investigation; however, their actual impact on SARS-CoV-2 viral transmission remains to be determined through experimental models [38,39].

Overall, according to the evidence that is currently available, cucurbitane-type triterpenoid glycosides from MC possess in vitro activity against HIV-1 and are expected to have inhibitory effects against the influenza virus. However, further in vivo and clinical research is required to determine their pharmacological significance and therapeutic potential.

### 3.2. Polysaccharides, Flavonoids, Saponins, and Sterols

MC contains a broad range of polysaccharides, flavonoids, saponins and sterols/fatty acids with antiviral properties (Figure 2).

The antiviral properties of polysaccharide fractions extracted from MC have been studied indirectly by evaluating their immunomodulatory properties. A recent work demonstrated that MC polysaccharides can modulate lymphocyte proliferation, cytokine production and macrophage activity in vitro, suggesting a host-directed antiviral mechanism in enhancing innate/adaptive responses rather than directly inactivating virus particles [30]. The protective effects of MC polysaccharide fractions were also demonstrated in an animal model of cyclophosphamide-induced immunosuppressed mice. The data specifically showed that high doses of MC polysaccharides (300 mg/kg) provided the strongest immunomodulatory effect, often restoring immune parameters (organ weight, cell proliferation and cytokine levels) to levels comparable to those in the normal healthy group [40]. However, it should be considered that the evidence demonstrating antiviral protection by MC polysaccharides in validated models is limited and often indirect (improved immune markers rather than reduced viral titers).

Flavonoids, saponins and sterols are widely recognized in plant pharmacology for their antiviral properties, which include blocking viral entry, inhibiting key enzymes for viral replication and exerting antioxidant and immunomodulatory effects. Specific MC flavonoids and saponins have been identified in phytochemical screens and some have been subjected to docking or indirect antiviral assays [6,31].

An in silico molecular docking analysis of MC leaf extract constituents indicated three flavonoids, quercetin-3-galactopyranoside, rutin and hyperin, with high theoretical binding scores against SARS-CoV-2 Mpro (known as 3C-like protease), suggesting potential antiviral activity of these derivatives and warranting further experimental validation [41]. Moreover, other bioactive compounds were identified as potential leads with predicted inhibitory activity against Mpro and the SARS spike glycoprotein–human ACE2 complex of SARS-CoV-2. These results suggest that this type of molecule needs further in vitro and in vivo investigation as a promising candidate against SARS-CoV-2 infection [42].

## 4. Antiviral Activity of Ribosome-Inactivating Proteins in *Momordica charantia*

Among all the phytochemicals identified in MC, RIPs are those supported by the most extensive experimental evidence for antiviral activity (Figure 2).

RIPs are a class of toxic enzymes widely distributed in the plant kingdom, and many plants producing RIPs have been used for centuries in traditional medicine [3]. RIPs are polynucleotide:adenosine glycosylases able to remove adenines from several intracellular polynucleotide substrates, thus causing the irreversible arrest of protein synthesis and consequent cell death [43,44]. Since viral replication relies on host protein synthesis machinery, RIP-mediated ribosome inactivation represents a broad-spectrum antiviral mechanism: by inhibiting host ribosomes, RIPs prevent infected cells from producing viral proteins, thus halting the viral life cycle. Several RIPs from different plant species, including well-studied ones like pokeweed antiviral protein (PAP) and trichosanthin, have demonstrated antiviral activity in vitro and in vivo against a wide range of viruses [33]. Beyond ribosomal inactivation, some RIPs have been shown to act more directly on viral genomes or transcripts; for instance, PAP can depurinate viral RNAs (or other polynucleotides), interfering with their replication, transcription or integration and further impairing viral protein synthesis [45,46,47].

The broad activity spectrum of RIPs supports their wide antiviral potential. Indeed, many RIPs have shown antiviral effects against a diversity of viruses: retroviruses (e.g., HIV), orthomyxoviruses (influenza), herpesviruses, flaviviruses, plant viruses and others [33].

Several RIPs have been isolated from the seeds and fruits of MC. The best-characterized RIPs are (i) momordica anti-HIV protein 30 kDa (MAP30); (ii) momorcharins (MMCs), i.e., α-momorcharin (α-MMC) and β-momorcharin (β-MMC); and (iii) momordin RIP (MCI). Many of these proteins have shown antiviral properties in in vitro and in vivo studies (Table 2). Despite the large number of studies on purified MC proteins, it is difficult to obtain a clear picture of the single protein effects because of the confusion in the nomenclature. This confusion has led to the same protein being designated by different names across various research groups. For example, α-MMC is also known as momordin I, M. charantia inhibitor (not to be confused with MCI), *M. charantia* isoRIP 3, momordin a or momordin (major isoform). In the same way, β-MMC is also called momordin II or *M. charantia* isoRIP 4 [48].

The first extensively characterized RIP from MC was MAP30, purified from seeds and fruit pulp [61]. Structural and biochemical analyses confirmed that MAP30 is a type I RIP of about 30 kDa, containing 263 amino acids with 30 basic residues [53]. The antiviral potential of MAP30, especially against HIV-1, was extensively documented in many seminal studies throughout the 1990s. Many of these papers are cited in a review by Fang and Ng, which defined the potential of MAP30 as a powerful and multi-target inhibitor of viral pathogenesis [62]. In HIV-1-infected cells, MAP30 inhibits reverse transcriptase activity and p24 core protein expression, suppressing viral replication with minimal host-cell toxicity. MAP30 also stopped the integration of the viral genome into the DNA of the host cell via several mechanisms, amongst them the irreversible relaxation of supercoiled viral DNA and the inhibition of viral integrase [62,63,64]. It is important to note that the narrow therapeutic window of RIPs remains a significant barrier to their clinical translation, as their potent inhibitory effects can lead to off-target cytotoxicity in more complex biological systems.

MAP30 showed inhibition of hepatitis B virus (HBV) DNA replication and HBs or HBe antigen secretion. The anti-HBV activity of MAP30 was studied in human hepatoma G2.2.15 cells, showing effective inhibition of HBV gene expression and a decrease in the genome replication of HBV DNA of intracellular replicative intermediates and of covalently closed circular DNA. MAP30 dose- and time-dependently inhibited the production of HBV. Low doses of MAP 30 were described to reduce viral antigen expression, whilst higher doses were effective in suppressing viral replication by altering the kinetics of replicative DNA intermediates [51].

MC leaf extracts showed only modest in vitro antiviral activity against human herpes virus 3 or varicella zoster virus (VZV), with inhibition observed at high concentrations (aqueous extract at 250 µg/mL and ethanolic extract at 125 µg/mL), indicating lower potency compared with other plant extracts and acyclovir. Overall, these results suggest that MC has limited anti-VZV efficacy and would require further optimization to be considered as an antiviral candidate [65].

In vitro studies showed antiviral activity of MAP30 against the herpes simplex virus (HSV); moreover, MAP30 was effective against an HSV-specific nucleoside analog acyclovir-resistant strain [62]. This effect could be of particular relevance for HIV patients, who are often resistant to acyclovir and in whom HSV infections are much more severe, frequent and life-threatening [66].

MAP30 also showed good antiviral activity against the dengue virus (DENV). The anti-DENV recombinant fusion protein PG1-MAP30-PLSN was obtained by linking the antiviral cationic peptides protegrin-1 (PG1) and plectasin (PLSN) with MAP30. The PG1-MAP30-PLSN protein considerably inhibited DENV protease (NS2B-NS3pro) with a half-maximal inhibitory concentration of 0.56–0.1 mM. In vitro assays showed considerable inhibition of the peptide-fusion protein against the binding and proliferating stages of DENV in the target cells. Moreover, the peptide-fusion protein protected DENV-challenged mice, leading to 100% survival at a dose of 50 mg/kg [50].

MAP30 and MCI potently inhibit SARS-CoV-2 replication in human lung cells, with IC_50_ values of around 0.2 µM and minimal cytotoxicity. A mutational analysis confirmed that the RNA N-glycosylase activity of these two RIPs is essential for their antiviral action, supporting their potential as robust candidates for therapeutic development against emerging coronavirus variants [52].

MMCs are RIPs isolated from the seeds and fruits of bitter melon, characterized by a basic single chain and present in two main isoforms. α-MMC has a molecular weight of approximately 30 kDa and a neutral sugar content of about 1.6%. β-MMC is a 29 kDa glycoprotein with a neutral sugar content of about 1.3%. Both MMCs lack hemagglutinating (lectin) activity and possess similar structural and biological properties, but are immunologically distinct [67].

The first studies conducted on the antiviral activity of α-MMC demonstrated that α-MMC inhibited HIV-1 III B-inducing C8166 syncytia formation and markedly reduced both the expression of the p24 core antigen and the numbers of HIV-antigen-positive cells in acutely but not chronically HIV-1-infected cultures [68].

At standardized concentrations (100 nM), α-MMC and β-MMC were shown to enhance the interaction between CD4 and gp120, a phenomenon reported for several RIPs, although this effect was not directly linked to antiviral efficacy. More importantly, both RIPs demonstrated potent inhibition of HIV-1 integrase, while exerting only weak suppression of reverse transcriptase and protease activities [54]. This suggests that MMCs’ antiviral contribution is tied predominantly to their ability to obstruct the post-entry steps of viral replication involving the integrase-mediated processing of viral DNA. The integrase inhibitory activity of MMCs aligns with the broader behavior of RIPs, which can target nucleic acid substrates beyond rRNA and induce lesions that impair key viral enzymes. These findings suggest that MMCs’ N-glycosylase activity or associated nucleic acid-damaging properties may underlie their ability to block integrase function.

The antiviral profile of α-MMC was also evaluated via dose- and time-dependent inhibition of HBV DNA synthesis. After 6 days of continuous treatment, α-MMC significantly reduced extracellular HBV DNA levels across all tested concentrations, with the strongest effect observed at 40 µg/mL. At this concentration, HBV DNA copies were reduced to approximately 6 × 10^5^ copies/mL, corresponding to about 58% inhibition compared to untreated controls. Moderate inhibition was also observed at 10 µg/mL (about a 39% reduction) and 2.5 µg/mL (about a 16% reduction), demonstrating a clear concentration–dose response. Importantly, the suppression of HBV replication occurred without substantial cytotoxicity at the tested antiviral concentrations. α-MMC displayed low toxicity toward HepG2.2.15 cells at doses up to 40 µg/mL, indicating that the antiviral effects are not attributable to non-specific cell death or loss of cell viability [56].

Direct antiviral activity, primarily demonstrated against HSV-1, is exhibited by α-MMC and MAP30. Both RIPs inhibited viral replication in infected Vero cells in a dose-dependent manner, significantly reducing HSV-1 glycoprotein antigen secretion. The reported IC_50_ values indicate potent antiviral effects, with MAP30 (0.47 μM) being more active than α-MMC (0.67 μM) and more effective than acyclovir in the same assay (IC_50_ 2.39 μM). Across all assays, MAP30 consistently showed stronger antiviral potency than α-MMC, mirroring previous observations that MAP30 is the most active antiviral RIP from MC [55].

## 5. Conclusions and Future Directions

The need for novel antiviral agents with a variety of mechanisms of action is highlighted by the emergence of resistant strains, chronic viral infections and recurrent epidemics of novel viruses. MC derivatives represent a very promising resource for the development of natural antiviral agents. Complex plant phytochemistry, particularly the activity of RIPs like MAP30 and MMCs, which target important post-entry steps of viral replication, determines MC potentiality. The theoretical potential of MC phytoconstituents was established mainly by computational studies. According to molecular docking studies, some MC constituents, principally triterpenoids and flavonoids, demonstrate strong binding affinities with main viral proteases, suggesting hypothetical mechanisms for inhibiting viral replication. Although molecular docking represents a useful preliminary tool for identifying the antiviral activity of MC-derived compounds, this approach has some limitations. In fact, in silico studies do not account for biological complexity, including host–virus interactions, intracellular viral replication, off-target effects or cytotoxicity. Furthermore, in silico predictions do not account for pharmacokinetic parameters such as absorption, bioavailability, metabolism and clearance, which are critical for clinical applicability.

In conclusion, MC emerges as a promising candidate for the prevention/treatment of viral diseases through a two-pronged strategy. Standardized MC extracts may represent a nutraceutical resource as dietary supplements to support immune health and provide broad-spectrum antiviral remedies, contributing to the prevention and treatment of viral infections. The plant bioactive properties can also be used in localized topical treatments, through pharmaceutical formulations such as transdermal patches or herbal gels, targeting cutaneous or mucosal viral infections. At the same time, MC represents a valuable source of bioactive compounds that could be further developed into specific antiviral agents after they have undergone rigorous pharmacological characterization and toxicity profiling. While the clinical utility of MC derivatives is currently limited by a narrow therapeutic index, the potential immunogenicity of protein components like RIPs, and a lack of standardized clinical data, MC remains a promising resource for antiviral drug discovery and nutraceutical development.

## Figures and Tables

**Table 1:** Principal constituents of *Momordica charantia* and reported antiviral activities.

Phytochemical Class	Representative Molecules	Plant Source	Antiviral Activity	References
Cucurbitane-type triterpenoid glycosides	Momordicosides, karavilosides,	Fruits, seeds, leaves, roots	Inhibition of viral replication and proteases; potential envelope interactions	[24,25,26,27,28]
Polysaccharides	MC polysaccharide fractions	Fruit pulp	Macrophage/cytokine modulation; host-directed antiviral support	[29,30]
Flavonoids/phenolics/saponins	Quercetin/kaempferol glycosides, catechins, gallic acid	Leaves, pericarp, fruits	Antioxidant/immunomodulatory; context-dependent interference with entry/replication of virus	[23,31]
Sterols/fatty acids	β-sitosterol, stigmasterol, linoleic/oleic acids	Seeds, fruits	Anti-inflammatory support during viral infection; modulation of immune responses	[11,32]
Ribosome-inactivating proteins (RIPs)	MAP30, α-momorcharin	Seeds, fruits	Inhibition of viral protein synthesis; reduced viral gene expression	[33,34,35]

**Table 2:** RIPs purified from *Momordica charantia* tested for antiviral activity.

RIP	Source	Mw (kDa)	Activity Against Animal Viruses	Activity Against Plant Viruses	Ref.
MAP30	Seeds/fruits	30	DENV, HBV, HIV-1, HSV, SARS-CoV-2		[49,50,51,52,53]
α-Momorcharin	Seeds	28.7	HBV, HIV-1, HSV	CMV, ChiVMV, TMV, TuMV	[54,55,56,57,58,59,60]
β-Momorcharin	Seeds	29	HIV-1		[54]
Momordin RIP (MCI)	Seeds	23	HSV, HPV; SARS-CoV-2		[52]

ChiVMV: chilli veinal mottle virus; CMV: cucumber mosaic virus; DENV: dengue virus; HBV: hepatitis B virus; HIV-1: human immunodeficiency virus 1; HPV: human papilloma virus; HSV: herpes simplex virus; SARS-CoV-2: severe acute respiratory syndrome coronavirus 2; TMV: tobacco mosaic virus; TuMV: turnip mosaic virus.

## Data Availability

No new data were created or analyzed in this study.

## Abbreviations

The following abbreviations are used in this manuscript:

α-MMC	α-Momorcharin
β-MMC	β-Momorcharin
ChiVMV	Chilli veinal mottle virus
CMV	Cucumber mosaic virus
DENV	Dengue virus
HBV	Hepatitis B virus
HIV-1	Human immunodeficiency virus 1
HPV	Human papilloma virus
HSV	Herpes simplex virus
MAP30	Momordica anti-HIV protein of 30 kDa
MC	*Momordica charantia*
MCI	Momordin
MMC	Momorcharin
PAP	Pokeweed antiviral protein
PG1	Protegrin-1
PLSN	Plectasin
RIP	Ribosome-inactivating protein
SARS-CoV-2	Severe acute respiratory syndrome coronavirus 2
VZV	Varicella zoster virus
